# Comparative analysis of diagnostic performance, feasibility and cost of different test-methods for thyroid nodules with indeterminate cytology

**DOI:** 10.18632/oncotarget.17220

**Published:** 2017-04-19

**Authors:** Salvatore Sciacchitano, Luca Lavra, Alessandra Ulivieri, Fiorenza Magi, Gian Paolo De Francesco, Carlo Bellotti, Leila B. Salehi, Maria Trovato, Carlo Drago, Armando Bartolazzi

**Affiliations:** ^1^ Department of Clinical and Molecular Medicine, Sapienza University, 00161 Rome, Italy; ^2^ Laboratory of Biomedical Research, Niccolò Cusano University Foundation, 00166 Rome, Italy; ^3^ Department of Oncological Science, Breast Unit, St Andrea University Hospital, 00189 Rome, Italy; ^4^ Operative Unit Surgery of Thyroid and Parathyroid, Sapienza University of Rome, St Andrea Hospital, 00189 Rome, Italy; ^5^ Department of Biopathology and Diagnostic Imaging, Tor Vergata University, 00133 Rome, Italy; ^6^ Department of Clinical and Experimental Medicine, Pad D 2° piano-AOU Policlinico “G. Martino”, 98125 Messina, Italy; ^7^ Department of Economics, Niccolò Cusano University, 00166 Rome, Italy; ^8^ Laboratory of Surgical and Experimental Pathology, St Andrea University Hospital, 00189 Rome, Italy; ^9^ Department of Oncology-Pathology, Cancer Center Karolinska Universitetssjukhuset Solna, S-17176 Stockholm, Sweden

**Keywords:** thyroid FNA cytology, indeterminate thyroid nodules, systematic review, meta-analysis, diagnostic performance

## Abstract

Since it is impossible to recognize malignancy at fine needle aspiration (FNA) cytology in indeterminate thyroid nodules, surgery is recommended for all of them. However, cancer rate at final histology is <30%. Many different test-methods have been proposed to increase diagnostic accuracy in such lesions, including Galectin-3-ICC (GAL-3-ICC), BRAF mutation analysis (BRAF), Gene Expression Classifier (GEC) alone and GEC+BRAF, mutation/fusion (M/F) panel, alone, M/F panel+*miRNA* GEC, and M/F panel by next generation sequencing (NGS), FDG-PET/CT, MIBI-Scan and TSHR *mRNA* blood assay.

We performed systematic reviews and meta-analyses to compare their features, feasibility, diagnostic performance and cost. GEC, GEC+BRAF, M/F panel+*miRNA* GEC and M/F panel by NGS were the best in ruling-out malignancy (sensitivity = 90%, 89%, 89% and 90% respectively). BRAF and M/F panel alone and by NGS were the best in ruling-in malignancy (specificity = 100%, 93% and 93%). The M/F by NGS showed the highest accuracy (92%) and BRAF the highest diagnostic odds ratio (DOR) (247). GAL-3-ICC performed well as rule-out (sensitivity = 83%) and rule-in test (specificity = 85%), with good accuracy (84%) and high DOR (27) and is one of the cheapest (113 USD) and easiest one to be performed in different clinical settings.

In conclusion, the more accurate molecular-based test-methods are still expensive and restricted to few, highly specialized and centralized laboratories. GAL-3-ICC, although limited by some false negatives, represents the most suitable screening test-method to be applied on a large-scale basis in the diagnostic algorithm of indeterminate thyroid lesions.

## INTRODUCTION

Follicular thyroid nodules with indeterminate pattern at fine needle aspiration (FNA) cytology are called in different ways, according to the different classification systems adopted. They are classified as thy3a in the presence of atypical features and as thy3f when a follicular neoplasm is suspected, according to the British Thyroid Association (BTA) [1]. They are defined as category III (Atypia of Undetermined Significance [AUS] or Follicular Lesion of Undetermined Significance [FLUS]) and IV (Follicular Neoplasm [FN] or Suspicious for a Follicular Neoplasm [SFN]), according to the Bethesda system [2]. In Italy, the Italian Society of Endocrinology (SIE), the Italian Thyroid Association (AIT), the Italian Association of Clinical Endocrinologists (AME) and the Italian Society for Anatomic Pathology and Cytology joint with the Italian Division of the International Academy of Pathology (SIAPEC-IAP) adopt the term Tir3A for low-risk and Tir3B for high-risk indeterminate lesions [3]. Independently of the classification system used, thyroid nodules classified in these categories represent the *gray zone* of conventional FNA-cytology [4, 5]. They are diagnosed in 15% - 30% of the total FNA cases and are currently referred for surgery more for diagnosis rather than for a real therapeutic necessity. Cancer prevalence in such indeterminate nodules varies according to the larger studies, performed in different Countries (Table 1).

**Table 1:** Cancer prevalence in thyroid nodules with indeterminate cytology

Author	Publication Year	Nodules with Indeterminate Cytology (resected)	Cancers at Histology (n.)	Cancers at Histology (%) (95% CI)
Davis [6]	1991	395	152	**38.5** (33.7-43.5)
Tuttle [7]	1998	103	22	**21.4** (13.9-30.5)
Raber [8]	2000	120	21	**17.5** (11.2-25.5)
Baloch [5]	2002	122	37	**30.3** (22.3-39.3)
Kim [9]	2003	215	102	**47.4** (40.6-54.3)
Sclabas [10]	2003	100	27	**27.0** (18.6-36.8)
Giorgadze [11]	2004	169	76	**45.0** (37.3-52.8)
Pu [12]	2006	303	87	**28.7** (23.7-34.2)
Wu [13]	2006	172	52	**30.2** (23.5-37.7)
Yassa [14]	2007	352	94	**26.7** (22.1-31.6)
Yang [15]	2007	378	100	**26.5** (22.1-31.2)
Oertel [16]	2007	391	103	**26.3** (22.0-31.0)
Mihai [17]	2009	201	57	**28.4** (22.2-35.1)
Banks [18]	2008	489	145	**29.7** (25.6-33.9)
Theoharis [19]	2009	129	48	**37.2** (28.9-46.2)
Sorrenti [20]	2009	603	106	**17.6** (14.6-20.9)
Asari [21]	2010	156	55	**35.3** (27.8-43.3)
Lubitz [22]	2010	144	16	**11.1** (6.5-17.4)
Rago [23]	2014	1,520	371	**24.4** (22.3-26.6)
**Pooled**		**6,062**	**1,671**	**27.6** (26.4-28.7)

Major studies (more than 100 cases) published in the literature concerning the occurrence of thyroid malignancy in indeterminate thyroid nodules. Data have been extrapolated by considering only tir3A/Tir3B/thy3a/thy3f/AUF/FLUS/FN/SFN cases. In all studies patients that received a cytological diagnosis of indeterminate thyroid nodules were surgically treated and histologically verified. Percentages of malignancy at histology, with corresponding 95% CI, are reported.

Pooled mean value is 27.6% (95% CI ranging from 26.4% to 28.7%). The lowest prevalence was reported in Boston, MA (11.1%, with 95% CI ranging from 6.5% to 17.4%) [22]. The highest prevalence was registered in South Korea (47.4%, with 95% CI ranging from 40.6% to 54.3%) [9]. In Italy the prevalence of malignancy in this type of nodules was 24.4% (95% CI ranging from 22.3% to 26.6%) and it didn't change over the time [23, 24], despite the increased incidence of thyroid cancer of 4.3% per year reported in the Italian general population [25, 26]. This means that about 70% of such indeterminate thyroid lesions that are referred to surgery are over-treated. Many different attempts have been made to ameliorate the accuracy of thyroid FNA-cytology by using clinical, ultrasonographic and scintigraphic features, but none of them proved to be accurate enough [27–29], even when a novel scoring system that combines and integrates the cytological information with the clinical and ultrasonographic risk factors of malignancy is used [30]. Several different test-methods have been proposed so far. They include new emerging molecular-based diagnostic tests, directly performed on thyroid FNA samples [31] or assay performed on peripheral blood [32], as well as nuclear medicine imaging tests, using tracers that are absorbed (“hot”) or excluded (“cold”) by the nodule, to detect thyroid malignancy [33–35] and expression analysis of putative protein tumor-associated markers, directly performed on thyroid FNA samples [31]. Both ATA Guidelines Task Force on Thyroid Nodules and Differentiated Thyroid Cancer [4] and NCCN Tumor Marker Task Force [36] suggested that the clinical utility of a molecular test should be founded in strong evidence proving that use of the marker “improves patient outcomes sufficiently to justify its incorporation into routine clinical practice” and it should not be intended to replace other sources of information or clinical judgment. The most extensively studied genetic marker is represented by a mutation of the BRAF gene. The specific mutation consists in a T1799A transversion resulting in a V600E amino acid substitution, with subsequent constitutive activation of BRAF kinase. The potential diagnostic and prognostic value of this genetic marker is proved by many studies [37, 38]. The diagnostic relevance of such marker is also demonstrated by the fact that it is included in all the composite molecular genetic panels proposed so far for recognizing thyroid malignancy in indeterminate thyroid nodules. Among the various thyroid cancer protein markers, Galectin-3 represents one of the most extensively studied. Galectin-3 is a multifunctional molecule involved in regulation of apoptosis [39, 40] whose potential role as a thyroid cancer marker was recognized by many studies since the first report published in 1995 [41]. A test-method based on Galectin-3 immunocytochemistry (GAL-3-ICC) analysis on thyroid FNA samples, named *ThyroTest*, has been developed and validated for clinical use in two large multicenter studies [42, 43] as well as in many others studies in different Countries [44].

The availability of so many different diagnostic tools for the preoperative characterization of thyroid nodules with indeterminate cytology urgently demands a comparative analysis of their diagnostic performance, feasibility, cost and effectiveness. Such a comparison, based on data collected in different clinical settings of various geographical and socio-economical contexts and involving diverse laboratory expertise, may be extremely challenging. For a more accurate comparative analysis the same indeterminate thyroid nodule population should be investigated by different test-methods. Recently, some attempts have been made to compare in the same nodule population some of the available test-methods [45–48]. The present extensive comparative analysis includes many different histopathological, genetic and imaging biomarkers and is aimed to provide to clinicians key information for a more cost-conscious clinical management of patients bearing indeterminate thyroid nodules. We considered the following test-methods: GAL-3-ICC, BRAF mutation analysis, GEC methods alone (*Afirma®* GEC by Veracyte) and in conjunction with BRAF mutation detection (*Afirma®* GEC + BRAF by Veracyte), thyroid cancer M/F panel (miR*Inform™* by Asuragen, and by Quest Diagnostics and *ThyGenX®* by Interpace Diagnostic), alone and in conjunction with *miRNA* GEC (*ThyGenX®* + *ThyraMIR™*) and thyroid cancer M/F panel analyzed by next generation sequencing (NGS) (*Thyroseq®* v.2 by CBLPath), [^18^F]-2-fluoro-2-deoxy-d-glucose–positron-emission-tomography/computed-tomography (FDG-PET/CT), [^99m^Tc]-methoxyisobutylisonitrile scintigraphy (MIBI-Scan) and TSHR *mRNA* blood assay. A systematic literature search was performed for each one of them. According to established criteria, the most representative studies published from 2000 to 2016 were selected. We focused our attention to analyze the cancer prevalence of the population examined, the technical methodology used, the feasibility, the cost and the diagnostic performance of each one of test-method included in the study. Data regarding the various indicators of diagnostic performance and of the cost for each one of them were used to perform a comparative statistical analysis.

## RESULTS

### Characteristics of the included studies

**Figure 1:**
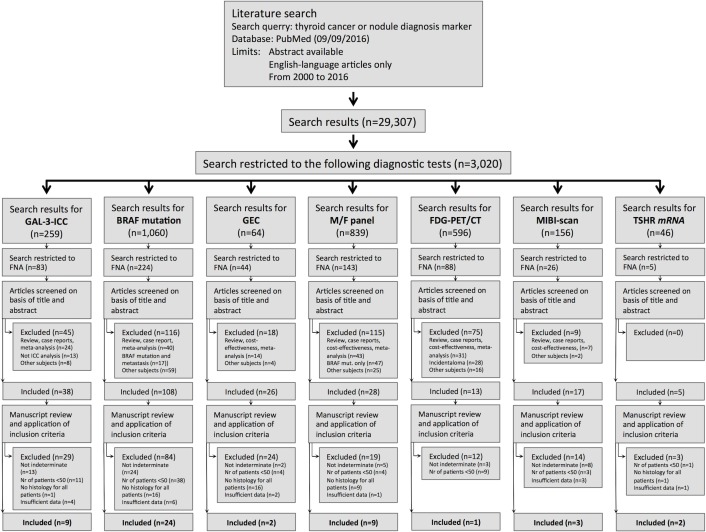
Flow of information of our systematic review for each test-method, through the four-phase flow, according to the PRISMA statement

A total of 45 different studies were selected on the basis of the criteria described in Materials and Methods section and following current protocols and guidelines [49]. The search strategy and the flow of information of our systematic review are reported in Figure 1. The features of the studies that met all the inclusion criteria are summarized in Table 2.

**Table 2:** Characteristic of the studies included in the comparative analysis

			Gene Expression Classifier (GEC)		Mutation/Fusion panel					
*Test-methods to Select Thy-3 nodules for surgery*	GAL-3-ICC	BRAF Mutation Analysis	GEC (Veracyte *Afirma®)*	GEC + BRAF (Veracyte *Afirma®* + BRAF)	Mutation/Fusion panel *(Asuragen miRInform™*, Quest Diagnostic & Interpace Diagnostic *ThyGenX®)*	Mutation/Fusion panel + miRNA GEC (Interpace Diagnostic *ThyGenX®/* *ThyraMIR™)*	Mutation/ Fusion panel by NGS *(Thyroseq* Ver. 2.0)	FDG- PET/CT	MIBI-Scan	TSHR *mRNA* Assay
**Bio-molecular Marker Type**	Protein *(Galectin-3)*	DNA *(1 gene, 1 codon)*	RNAs *(167 genes)*	RNAs & DNAs *(167 genes)* + BRAF mutation	RNAs and DNAs *(4 genes, 14 SNPs and 3 chromosome rearrangements)*	RNAs and DNAs *(4 genes, 14 SNPs & 3 chromosome rearrangements) + 10 miRNA*	RNAs & DNAs *(13 genes and 42 gene fusions)*	Glucose uptake	Sesta-MIBI uptake	TSHR mRNA
**Method**	Immuno- CytoChemistry	BRAF (V600E) mutation analysis	Gene Expression Classifier	Gene Expression Classifier + BRAF (V600E) mutation analysis	RT-PCR, Fluorescence Melting Curve, Luminex, Sanger sequencing, Pyrosequencing	RT - qPCR Luminex	Next Generation Sequence (NGS)	FDG- PET/CT	Thyroid Scintigraphy (visual analysis)	qRT -PCR Blood Assay
**Patients and samples recruitment**	Pooled from 9 studies	Pooled from 24 studies	Multicenter (49, USA)		Multicenter (USA, Denmark, Italy) Pooled from 8 studies	Multicenter (USA)		1 Center (USA)	Pooled from 3 studies (Italy)	Pooled from 2 studies (USA)
**Laboratory for Testing**	9 Centers (Europe and Chile)	24 Centers (Europe, Canada, USA, China, Korea)	1 Center (USA)		1 Center (USA)			1 Center (USA)	3 Centers (Italy)	1 Center (USA)
**Total number of cases**	1,266	2,625	210	165	1,141	109	143	51	217	114
**Cancer** **Prevalence (%) (95% CI)**	33 (30.9-36.2)	45 (42.8-46.6)	24 (18.6-30.7)	27 (20.0-34.1)	24 (21.7-26.7)	32 (23.5-41.7)	27 (20.2-35.3)	20 (9.82-33.1)	29 (23.1-35.6)	44 (34.6-53.5)
**True Positive (TP) eqv. with hit**	351 (27.7%)	474 (18.1%)	46 (21.9%)	39 (23.6%)	141 (12.4%)	31 (28.4%)	35 (24.5%)	8 (15.7%)	42 (19.4%)	35 (30.7%)
**True Negative (TN) eqv. with correct rejection**	716 (56.6%)	1,451 (55.3%)	82 (39.0%)	60 (36.4%)	805 (70.6%)	63 (57.8%)	97 (67.8%)	25 (49.0%)	129 (59.4%)	52 (45.6%)
**False Positive (FP) eqv. with false alarm**	127 (10.0%)	4 (0.1%)	77 (36.7%)	61 (37.0%)	61 (5.3%)	11 (10.1%)	7 (4.9%)	16 (31.4%)	25 (11.5%)	12 (10.5%)
**False Negative (FN) eqv. with miss**	72 (5.7%)	696 (26.5%)	5 (2.4%)	5 (3.0%)	134 (11.7%)	4 (3.7%)	4 (2.8%)	2 (3.9%)	21 (9.7%)	15 (13.2%)
**Reference**	[43, 78, 80-86]	[46, 51-53, 56-58, 63-79]	[60]	[61]	[51-58]	[54]	[59]	[62]	[34, 35, 46]	[32, 50]

Comparison of the seven different test-methods and their variants, proposed for the same diagnostic purpose in thyroid nodules with indeterminate cytology. The type of bio- molecular marker, the method used, the total number of cases examined and the type of the study are also indicated. For each test-method the cancer prevalence, with 95% CI, and the results are reported.

Selected studies were performed in different geographical areas and in different Institutions. The specific features and test outcome, according to the gold standard of final histology, are reported. Patients were enrolled either in Academic Medical Centers, in University Hospitals or in private companies. In some studies, the test was performed in two subsequent periods, by the same Academic Medical Center in U.S.A. [32, 50]. In others, patients were enrolled in thyroid Centers of different Countries, such as U.S.A. [51–54, 59], Denmark [55], Canada [56], China [57] and Italy [58], and the samples were referred to a single central laboratory for molecular analysis. In other studies the same population of thyroid nodules, analyzed with one test was also subjected to another additional test [54, 61]. There are differences regarding also the total number of cases examined in these studies. In three test-methods, namely the BRAF mutation analysis [46, 51–53, 56–58, 63–79], the M/F panel [51–58] and the GAL-3-ICC [43, 78, 80–86], more than 1,000 cases from different studies were pooled together. In the case of the other six test-methods, the GEC alone [60], the GEC plus BRAF [61], the M/F panel plus *miRNA* GEC [54], the M/F panel by NGS [59], the TSHR *mRNA* [32, 50] and MIBI-scan [34, 35, 46], more than 100 cases were retrieved and pooled together. Finally, in the case of FDG-PET/CT [62] only slightly more than 50 cases were analyzed from one single study. The cancer prevalence reported in the selected studies ranged from 20% to 44%, and was in line with that reported in the literature for indeterminate nodules (Table 1).

### Assessment of study heterogeneity

**Figure 2:**
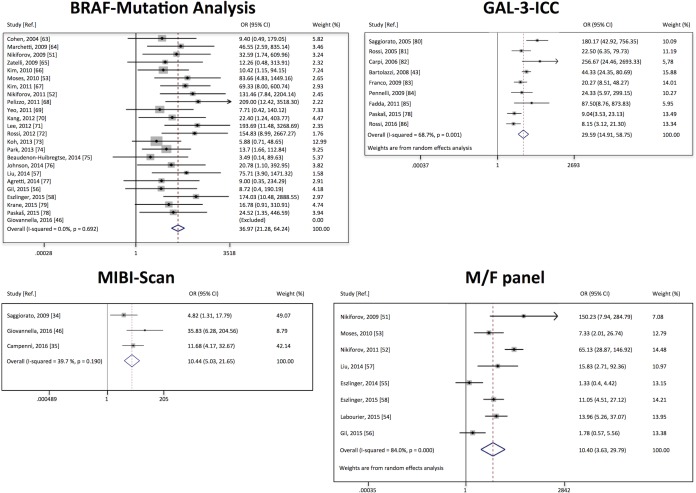
Forest plots of individual studies and pooled odds ratio of the four test-methods for which more then 2 studies were selected from the systematic literature search For each study the 95% confidence interval (95% CI) and the weight (%) were also reported. The open diamond at the bottom of the graph shows the average effect size of the examined studies.

In 4 test-methods, namely the BRAF mutation analysis, GAL-3-ICC, MIBI-Scan and M/F panel, the systematic literature search, based on the established criteria, allowed us to select multiple studies, suitable to perform a meta-analysis. The graphical representations of these four meta-analyses are visualized in Figure 2. In the forest plot of GAL-3-ICC and MIBI-Scan no overlapping of the vertical line, corresponding to odds ratio = 1, with the confidence intervals of all studies was observed, indicating that all these studies are significant at 5% level. The same vertical line (odds ratio = 1) overlaps the confidence intervals of 7 out of 24 studies in BRAF mutation analysis, and of 2 out of 8 studies in M/F panel, indicating that these studies were less significant. In the forest plot of BRAF mutation, the last study [46] was excluded from the analysis because statistical calculations were not feasible. In that study, in fact, no positive results, either true or false, were reported. The I-squared (I^2^) was measured for the selected studies of each test-method to evaluate study heterogeneity. The overall I^2^ was 0% in the BRAF group, indicating that inconsistency across studies may not be important. The overall I^2^ was 39.7% in MIBI-Scan group and 68.7% in GAL-3-ICC group, indicating occurrence of moderate heterogeneity. Finally, The overall I^2^ was 84% in the M/F panel group, suggesting occurrence of substantial heterogeneity.

### Tests classified according to their ability to exclude (rule-out) malignancy

The ability of a negative diagnostic test-method to exclude malignancy in a thyroid nodule with indeterminate cytology would be extremely useful in surgical decision-making, contributing to promptly identify those benign lesions that can be directed to follow-up. We therefore classified the different diagnostic test-methods analyzed based on this ability (Table 3).

**Table 3:** Test methods classified according their ability in ruling-out or ruling-in malignancy, to their diagnostic performance and their cost

			Gene Expression Classiifer (GEC)		Mutation/Fusion panel					
*Test-methods to Select Thy-3 nodules for surgery*	GAL-3- ICC	BRAF Mutation Analysis	GEC (Veracyte *Afirma*®)	GEC + BRAF (Veracyte *Afirma*® + BRAF)	Mutation/Fusion panel (*Asuragen* miR*Inform*™, Quest Diagnostic & Interpace Diagnostic *ThyGenX*®)	Mutation/Fusion panel + *miRNA* GEC (Interpace Diagnostic *ThyGenX*®/ *ThyraMIR*™)	Mutation/Fusion panel by NGS (*Thyroseq* Ver. 2.0)	FDG-PET/ CT	MIBI-Scan	TSHR *mRNA* Assay
**A-Rule-out cancer**										
**Sensitivity (%) (95% CI)**	83 (79.8–85.8)	41 (39.9–40.7)	90 (79.1–96.3)	89 (76.3–95.6)	51 (46.9–55.2)	89 (75.6–96.0)	90 (78.4–96.2)	80 (47.3–96.4)	67 (56.2-75.6)	70 (59.3-78.6)
**Negative Predicted value (NPV) (%)**	91	68	94	92	86	94	96	93	86	78
**Cancer risk rate in negative test (%)**	9	32	6	8	14	6	4	7	14	22
**False Negative Rate (FNR) (%)**	17	59	10	11	49	11	10	20	33	30
**Negative Likelihood Ratio (LR-) (95% CI)**	0.2 (0.16–0.24)	0.6 (0.59–0.6)	0.19 (0.07–0.44)	0.23 (0.08–0.52)	0.52 (0.47–0.58)	0.13 (0.04–0.31)	0.11 (0.04–0.24)	0.33 (0.06–0.99)	0.4 (0.28–0.55)	0.37 (0.24–0.56)
**B-Rule-in cancer**										
**Specificity (%) (95% CI)**	85 (83.3–86.3)	100 (99.3–100.0)	52 (48.0–53.5)	50 (45.1–52.1)	93 (91.6–94.2)	85 (79.0–88.6)	93 (89.0–95.7)	61 (53.0–65.0)	84 (79.5–87.4)	81 (72.9–88.0)
**Positive Predicted value (PPV)**	73	99	37	39	70	74	83	33	63	74
**False Positive Rate (FPR) (%)**	27	0	48	50	7	15	7	39	16	19
**Positive Likelihood Ratio (LR+) (95% CI)**	6 (4.79–6.29)	147 (53.8–461.5)	2 (1.52–2.07)	2 (1.39–2.61)	7 (5.58–9.53)	6 (3.60–8.45)	13 (7.14–22.36)	2 (1.00–2.75)	4 (2.74–6.02)	4 (2.18–6.53)
**C–Diagnostic Performnce**										
**Accuracy (%) (95% CI)**	84 (82.2–86.2)	73 (72.8–73.5)	61 (55.6–63.9)	60 (53.4–63.7)	83 (80.8–84.8)	86 (77.9–91.0)	92 (86.1–95.8)	65 (51.9–71.1)	79 (72.7–84.0)	76 (66.9–83.9)
**F1 score (%)**	78	58	53	54	59	81	86	47	65	72
**Diagnostic Odds Ratio (DOR)**	27	247	10	8	14	44	121	6	10	10
**D–Single Test Cost**										
**Single Test cost (USD)**	113	97.45	3,200	3,675	2,250	3,300	3,200	1,132	1,648	300
*Reference*	[43, 78, 80–86]	[46, 51–53, 56–58, 63–79]	[60]	[61]	[51–58]	[54]	[59]	[62]	[34, 35, 46]	[32, 50]

A. Tests classified according to their ability to exclude malignancy (rule–out tests). B. Tests classified according to their ability to detect malignancy (rule–in tests). C. Tests classified according to their diagnostic performance. D. Tests classified according to their costs in USD.

For each test the 95% CI of sensitivity, specificity, accuracy, LR+ and LR– is reported in brackets.

The best rule-out indicator is the sensitivity (“positivity in disease”) that refers to the proportion of subjects who have the target condition (malignancy at histology) and gives a positive test result. It corresponds to a high NPV and a low FNR. The present comparative analysis indicates that among all test-methods considered M/F panel by NGS, GEC alone, GEC + BRAF and M/F panel + *miRNA* GEC showed the highest sensitivities (90%, 90%, 89% and 89% respectively), the highest NPV (96%, 94%, 92% and 85% respectively), and the lowest FNR (10%, 10%, 11% and 11% respectively). These four test-methods appear, indeed, to be the most reliable ones for cancer exclusion (best rule-out methods). GAL-3-ICC and FDG-PET/CT were both characterized by high sensitivity (83% and 80% respectively), high NPV (91% and 93% respectively), with a low, but significant FNR (17% and 20% respectively) and cancer risk in negative lesions (9% and 7% respectively). TSHR *mRNA* blood assay, and MIBI-Scan showed a lower sensitivity (70% and 67% respectively), a lower NPV (78% and 86% respectively) and a higher FNR (30% and 33% respectively). The BRAF mutation analysis showed the lowest sensitivity among all tests (41%), the lowest NPV (68%) and the highest FNR observed (59%). The diagnostic performance of this test-method in excluding malignancy didn't improve much even if it was integrated with the analysis of a panel of thyroid specific M/F gene alterations. The use of M/F panel, in fact, yields only a slight increase in sensitivity (51%), of NPV (86%), a slightly reduced, but still elevated FNR (49%) as well as a high cancer risk in negative lesions (14%). Therefore, BRAF Mutation analysis and M/F panel are not adequate to efficiently exclude malignancy (worst rule-out methods).

### Tests classified according to their ability to detect (rule-in) malignancy

The ability of identifying malignancy among indeterminate thyroid nodules represents an important clinical achievement. Surgical decision based on a good rule-in test may lead clinicians to selectively refer to surgery only thyroid cancers, reducing the over-treatments of benign lesions and consequently the social costs. The test-methods were then classified according to this ability (Table 3). The best rule-in indicator is the specificity (“negativity in health”) that refers to the proportion of subjects without the target condition (malignancy at histology) and gives negative test results. It corresponds to high PPV and low FPR. There are many test-methods that proved to be good in detecting malignancy among the indeterminate thyroid follicular lesions. The best rule-in method is the BRAF mutation analysis. It showed an absolute specificity (100%) an almost absolute PPV (99%), with no occurrence of false positives (FPR = 0%). Many test-methods were characterized by a very high specificity (≥ 85%), namely the three variants of M/F panel-based test-methods (alone, + *miRNA* GEC and by NGS) and the GAL-3-ICC. They showed also a high PPV value (70%, 74%, 83% and 73% respectively) and a low FPR (7%, 15%, 7% and 27% respectively) (Table 3). Adjunction of the *miRNA* GEC to the M/F panel didn't seem to increase the ability of this method in identifying malignancy and the combination of these two methods, compared to the M/F panel alone displayed a slightly reduction in specificity (85%) and moderate increase in NPV (74%), with a higher FPR (15%). The MIBI-Scan and the TSHR *mRNA* showed a lower specificity (84% and 81% respectively), with a lower PPV (63% and 74% respectively), and a rather higher FPR (16% and 19% respectively). The FGD-PET/CT was characterized by a very low specificity (61%), the lowest PPV observed (33%) and a very high FPR (39%). GEC method, alone or in combination with BRAF mutation detection, didn't show a good rule-in performance, with the lowest specificity (52% and 50% respectively), a rather low PPV (37% and 39% respectively) and with the highest FPR (48% and 50% respectively). Both GEC alone and GEC + BRAF mutation analysis, therefore, appear to be not reliable for cancer detection (worst rule-in tests).

### Tests classified according to their combined ability to detect (rule-in) and to exclude (rule-out) malignancy

**Figure 3:**
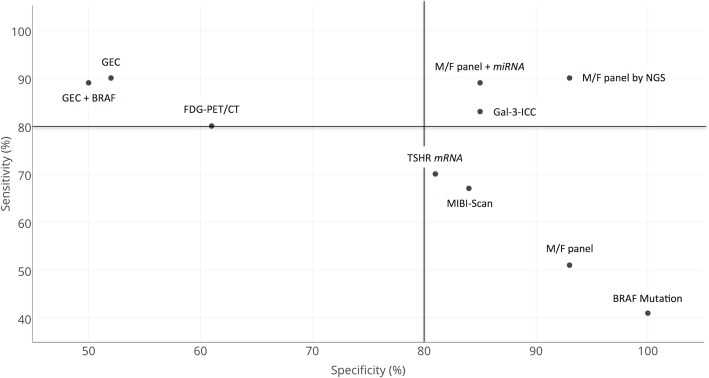
Comparative two-dimensional scatterplot diagram of sensitivity and specificity of each test-method Arbitrary cut-off lines at 80% of specificity and at 80% of sensitivity were included in the diagram.

Sensitivity and specificity of all test-methods analyzed were graphically expressed in a two-dimensional scatterplot diagram (Figure 3). FDG-PET/CT and GEC alone or in conjunction with BRAF mutation analysis, all characterized by high sensitivity (upper part of the diagram), showed a rather low specificity (left part of the panel). Many different test-methods reached very high specificity and are consequently plotted at the very right extremity of the diagram. However, they showed different levels of sensitivity. In the cases of the BRAF mutation analysis an absolute specificity (right-most side of the diagram) was associated with a rather low sensitivity (lowest part of the diagram). The M/F panel alone also showed a combination of high specificity (right extremity of the diagram) and low sensitivity (lower part of the diagram). The MIBI-Scan and TSHR *mRNA* were plotted in the lower part of the diagram, with their low sensitivities, combined with slightly better specificities. The adjunction of the *miRNA* GEC analysis to the M/F panel or its analysis by NGS greatly improved the sensitivity of this method and contributed to locate these two methods in the upper right part of the diagram. The M/F by NGS reached the best combination of sensitivity and specificity among all the test-methods analyzed. GAL-3-ICC showed equally good levels of sensitivity and specificity and is plotted in the most favorable upper right part of the diagram.

### Tests classified according to their likelihood ratios

**Figure 4:**
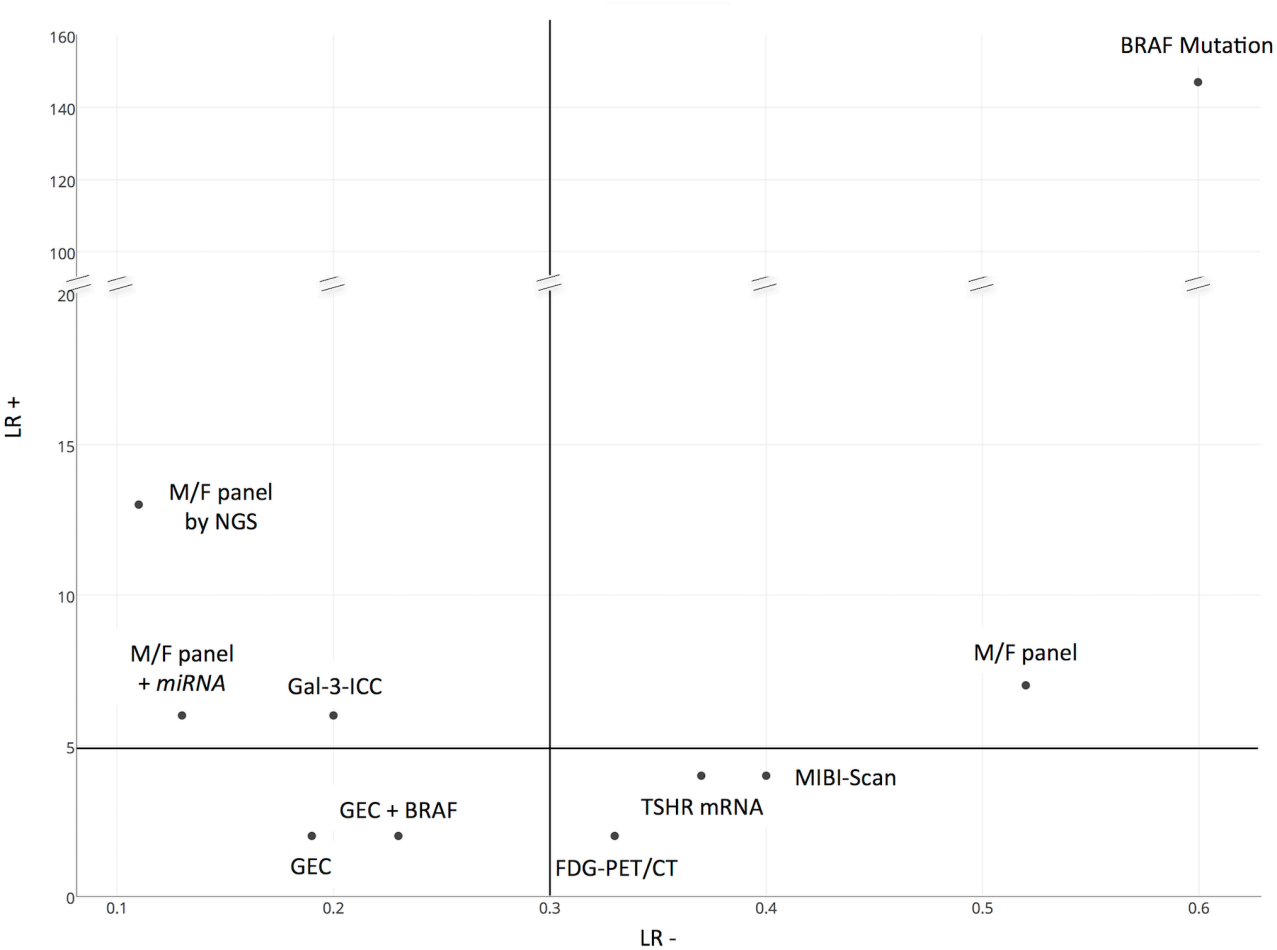
Comparative two-dimensional scatterplot diagram of LR+ and LR- of each test-method (LR+ Positive Likelihood Ratio, LR- Negative Likelihood Ratio). Arbitrary cut-off lines at 5 of LR+ and at 0.3 of LR- were included in the diagram.

In the decision process among competing diagnostic test-methods in a clinical application the use of positive and negative likelihood ratios, rather than simple value of sensitivity and specificity, as measures of diagnostic ability has been recommended [87]. Likelihood ratios have a number of useful properties, including the fact that they do not vary in different populations or settings and are independent of prevalence of the disease [88]. They are generally considered one of the best ways to measure and express diagnostic accuracy [89]. Larger values of LR+ and smaller values of LR- indicate greater diagnostic ability (discrimination ability). Therefore, when choosing a diagnostic test, one would prefer those with LR+ as high as possible and, simultaneously, with LR- as low as possible [87]. Data concerning LR+ and LR- values of the different test-methods are reported in Table 3 and visualized in Figure 4. Usually a value of LR+ >5 indicates that the test result has a moderate/large effect on increasing the probability of disease, while value of LR+ <5 indicates a small effect on increasing the probability of disease. On the other side, an LR- <0.3 indicates that the result has a large/moderate effect on decreasing the probability of disease presence, while a value of LR- >0.3 indicates a small effect on decreasing disease probability. The present comparative analysis indicates that some test-methods such as FDG-PET/CT, MIBI-Scan and TSHR *mRNA*, showed the unfavorable combination of very low LR+ and a high LR- and are plotted on the lower right part of the diagram. The test-methods that showed the most favorable combination of high LR+ and low LR- are the M/F panel by NGS, the M/F panel + *miRNA* GEC and the GAL-3-ICC, all plotted in the left upper part of the diagram. The two test-methods that were characterized by high LR+ and high LR- are BRAF mutation and M/F panel alone. In particular, the BRAF mutation was the method with the highest value of LR+ (out of scale in the diagram), combined, however, with the highest LR- value. Finally the GEC method alone and GEC + BRAF showed a low LR- combined with a low LR+.

### Tests classified according to their diagnostic performance

Three different parameters, all related to the ability to discriminate between benign and malignant thyroid nodules were measured, namely the accuracy, the F1 score and the DOR (Table 3). The test-method with the best accuracy is the M/F panel by NGS (92%), followed by M/F + *miRNA* GEC (86%), by GAL-3-ICC (84%) and by M/F panel alone (83%). The diagnostic accuracy of the remaining test-methods, namely the MIBI-Scan (79%), the TSHR *mRNA* (76%), the BRAF mutation analysis (73%), the FDG-PET/CT (65%), the GEC (61%) and the GEC+ BRAF (60%) were the lowest among all test-methods considered. We then classify the test-methods according to their F1 score, which equally weights recall (ratio of true positives to all actual positives) and precision (ratio of true positives to all predicted positives) and illustrates the overall accuracy of a test. According to this calculation, M/F panel by NGS and M/F panel + *miRNA* GEC were the two best test-methods among all. They showed, in fact, an F1 score of 86% and 81% respectively. GAL-3-ICC ranked among the top ones (78%), followed by the TSHR *mRNA* (72%) and the MIBI-Scan (65%). The M/F panel alone (59%), the BRAF mutation analysis (58%), both GEC test-methods, alone (53%) or + BRAF (54%), and the FDG-PET/CT (47%) showed the lowest F1 score among all. When test-methods were analyzed using the DOR we observed that the best value was obtained by BRAF mutation analysis (247), followed by M/F panel by NGS (121) and by M/F panel + *miRNA* GEC (44). GAL-3-ICC showed a good DOR (27). A lower DOR was measured for M/F panel alone (14), GEC alone (10), TSHR *mRNA* (10) and MIBI-Scan (10), while the lowest values were observed for GEC + BRAF (8) and FGD-PET/CT (6).

### Tests classified according to their cost, effectiveness and feasibility

**Figure 5:**
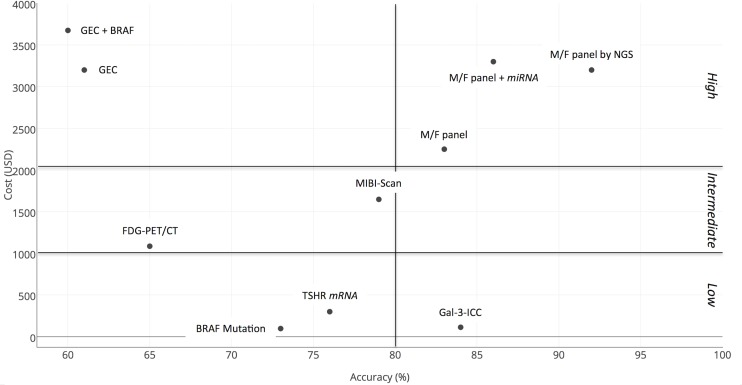
Comparative two-dimensional scatterplot diagram of cost and accuracy of each test-method Arbitrary cut-off lines at 80% of accuracy and at 1,000 and 2,000 USD were included in the diagram.

**Figure 6:**
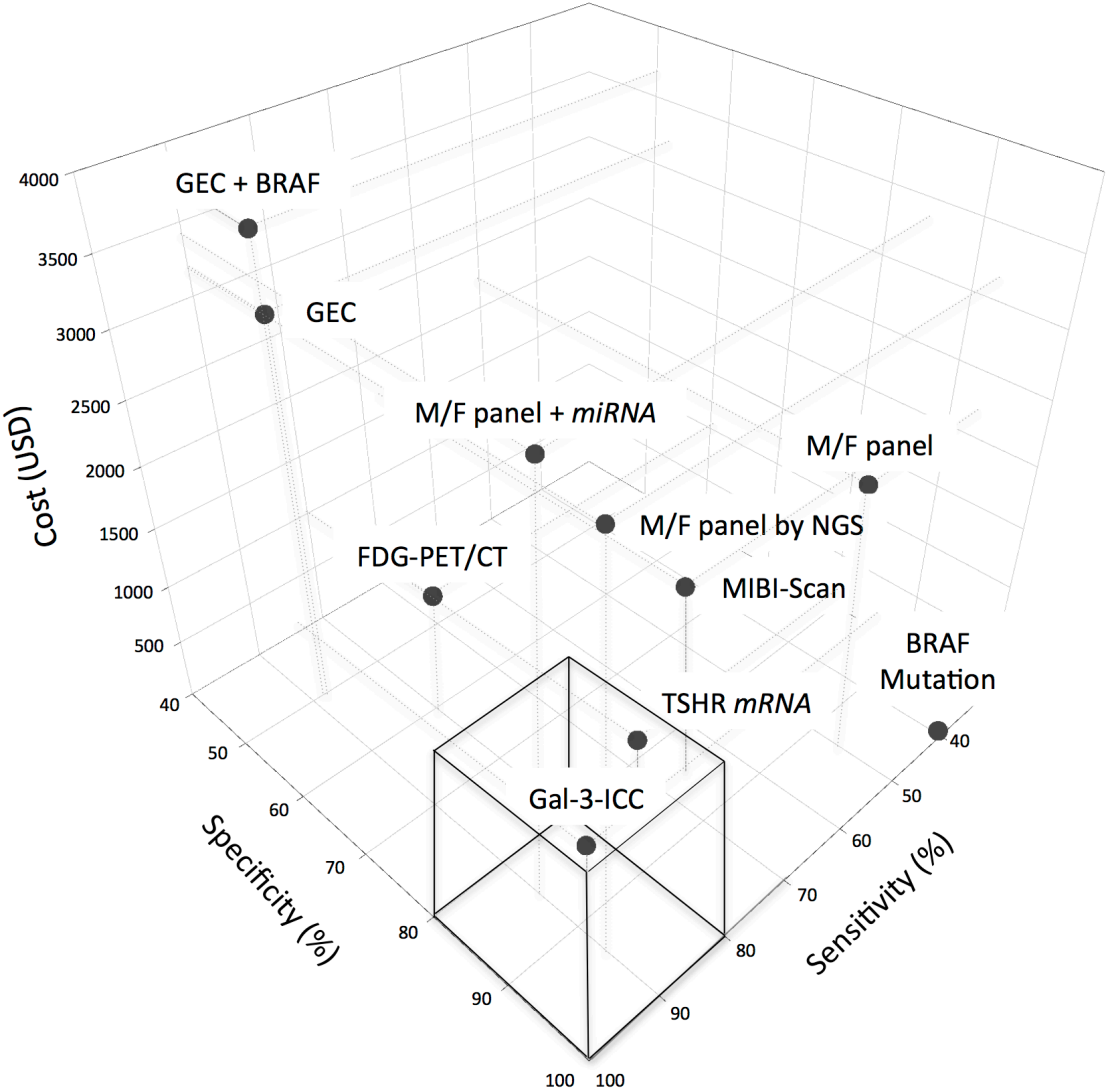
Comparative three-dimensional scatterplot diagram of cost, sensitivity and specificity of each test-method Arbitrary cut-off lines at 80% of sensitivity, at 80% of specificity and at 1,500 of single test cost, expressed in USD, were included in the diagram.

In the effort to control expenses we focused our attention not only on the possible benefits, practical feasibility and the diagnostic value of each test-method but also on the single test cost and, hence, the estimated costs for the community. Costs were expressed as 2016 USD (1 USD = 1,1306 Euro) [90]. A comparative analysis of the costs is shown in Table 3. All molecular based test-methods require the use of sophisticated instruments and reagents by specialized physicians in centralized well-equipped molecular laboratories and that is the reason why they are very expensive. In particular, the cost of each single GEC test was the highest one (3,200 USD/test) and the cost for a single M/F panel-based was 2,250 USD/test for Quest Diagnostic, 1,675 USD/test for *ThyGenX®* alone and 4,975 USD/test for the combination of *ThyGenX®* + *ThyraMIR™*. Compared to these molecular approaches, GAL-3-ICC is by far one of the cheapest, with its cost of 113 USD/test. Moreover GAL-3-ICC analysis is easy to be performed in any clinical context, in which a conventional surgical pathology laboratory is equipped to provide an immuno-cyto/histochemistry service. This assay integrates the diagnostic performance of conventional thyroid FNA-cytology and use the same cell substrates, which can be morphologically classified. Methods and reagents for GAL-3-ICC have been standardized for clinical use [91]. TSHR *mRNA* blood assay and BRAF mutation analysis are also cheap with their costs of 97.45 USD/test and 300 USD/test respectively. Unlike the other test-methods, TSHR *mRNA* blood assay doesn't require an FNA sampling of the thyroid nodule. The average cost of FDG-PET/CT is 1,132 USD/test [92] and it is similar to that for MIBI-Scan (1,648 USD/test) [48]. Both these procedures can only be performed in a highly specialized radiology and nuclear medicine Units, and they expose patients to radiations, that can be reduced if only the thyroid bed is scanned. However, for these reasons they are not recommended as routine screening methods. The comparative combined analysis of cost and effectiveness of the test-methods included in the study was conducted using both the two-dimensional (Figure 5) and the three-dimensional scatterplot diagrams (Figure 6). In the two-dimensional diagram the most accurate test-methods are plotted on the right part and the cheapest on the lower part of the diagram. GAL-3-ICC is the only test method to be plotted in the most favorable lower right part of the diagram, showing the best combination of high accuracy (84%) and low cost (113 USD/test). Cost and effectiveness of this procedure is optimal and, therefore, it represents a suitable screening method for the preoperative characterization of indeterminate thyroid nodules on a large-scale basis. BRAF mutation analysis and TSHR *mRNA* blood assay also ranked as cheap test-methods (97.45 USD/test and 300 USD/test respectively), but they showed lower accuracy rate (73% and 76% respectively) and they are, therefore, both plotted in the left lower part of the diagram. The three methods based on M/F panel analysis were located in the upper right part of the diagram, indicating an excellent accuracy but a high cost. Among them the one that is based on the NGS technique showed the best accuracy (92%), but its cost was rather high (3,200 USD/test). The two test-methods based on GEC analysis are both located in upper left part of the diagram, indicating a rather unsatisfactory combination of low accuracy and high cost, compared to the other test-methods analyzed. FDG-PET/CT and MIBI-Scan showed a combination of intermediate values of both accuracy (65% and 79% respectively) and cost (both tests > 1,000 USD and < 2,000 USD). To better analyze the effectiveness and cost sensitivity, specificity and cost of each single test was visualized on a three-dimensional scatterplot diagram (Figure 6). The diagram indicates that, as previously observed, the two molecular test-methods characterized by very high sensitivity, namely GEC alone and GEC + BRAF, do not combine this favorable feature with a correspondingly high specificity and, in addition, they are among the most expensive ones. For this reason they are plotted in the left/posterior/high sector of the 3D diagram. BRAF mutation, characterized by absolute specificity and low sensitivity, is one of the cheapest ones and is, therefore, plotted in right/anterior/low sector of the 3D diagram. M/F panel based test-methods, especially the one analyzed by NGS and the M/F panel + *miRNA* CEG, reached an excellent combination of very high sensitivity and very high specificity. However, the clinical use of all these molecular-based test-methods is hampered by its very high cost. FDG-PET/CT and MIBI-Scan are both characterized by a medium cost of each single exam. However, MIBI-Scan showed a slightly better specificity, while FDG-PET/CT was characterized by a slightly better sensitivity. The test-method that showed the best combination of high sensitivity, high specificity and low cost is the GAL-3-ICC, characterized, in fact, by good specificity (85%) and sensitivity (83%), combined with a very low cost (113 USD). This test-method, the only one that is plotted in the anterior and lower part of the 3D diagram, appears to be suitable to be chosen as a screening test on large-scale basis.

### Proposed diagnostic algorithm for indeterminate thyroid nodules

**Figure 7:**
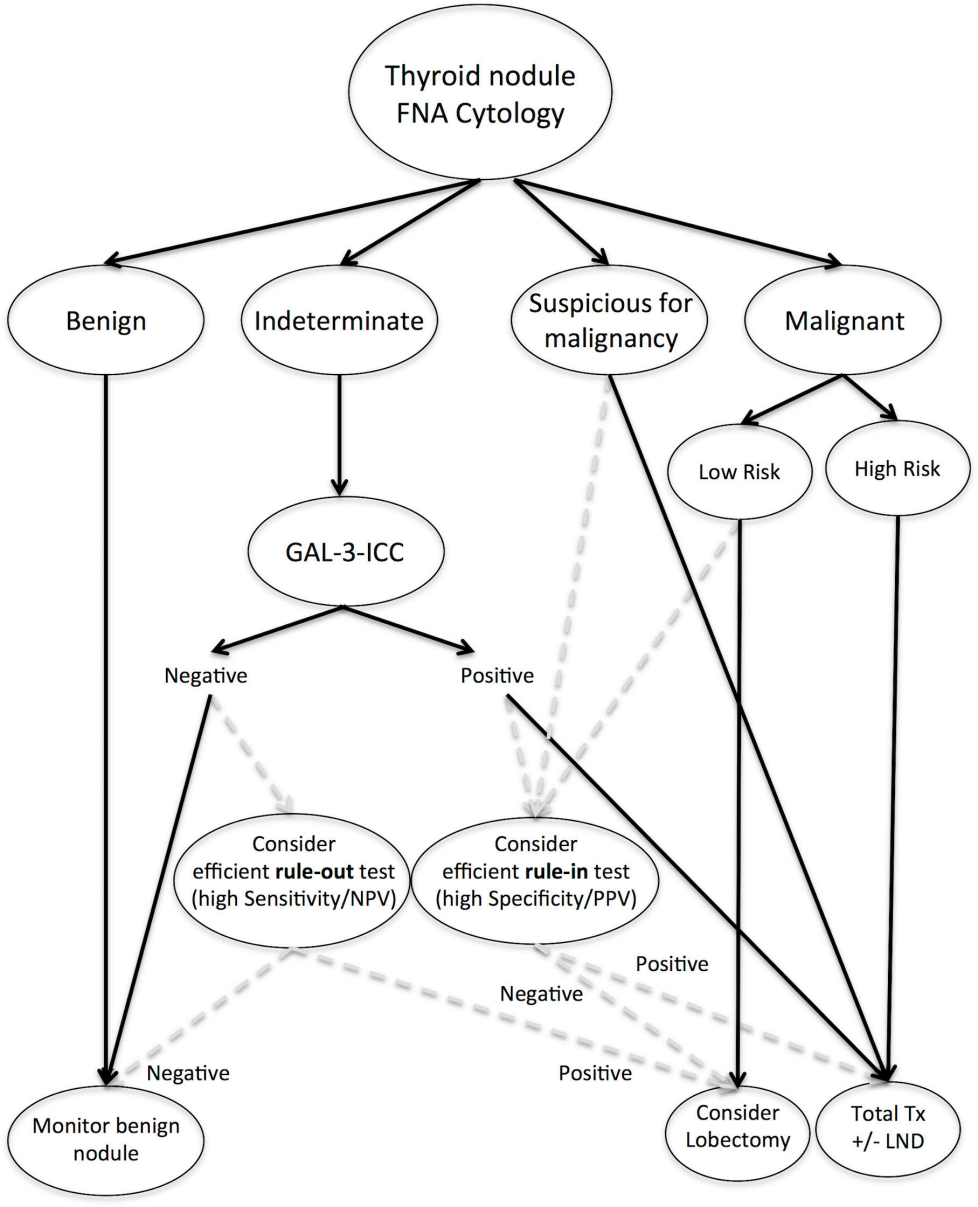
Algorithm for management of thyroid nodules

Based on the results of our comparative analysis we propose an algorithm that includes cytology and other ancillary test-methods in the management of patients with thyroid nodules (Figure 7). The advantage of this algorithm relies in the reduction of unnecessary surgery by means of a first level, low-cost test-method, which can be integrated, in selected cases, with a second-level high-cost molecular-based test-method. In fact, on the basis of our comparative data, we believe that patients with indeterminate thyroid nodules should be initially considered for GAL-3-ICC, a low-cost procedure that proved to be sufficiently highly sensitive and specific. Thyroid nodules with indeterminate cytology that were positive at GAL-3-ICC analysis should be considered for surgery, while GAL-3-ICC negative ones can be monitored by repeated ultrasonographic evaluation, without surgery, as recently suggested [93]. Molecular-based test-methods should be considered as second-line tests, appropriate in high-income Countries where highly specialized molecular genetics laboratory are available. In GAL-3-ICC-positive nodules the use of a more efficient rule-in test, such as M/F panel by NGS, would be useful to confirm malignancy and to better plan the extent of thyroidectomy. Conversely, in GAL-3-ICC-negative nodules the use of a more efficient rule-out test, such as GEC, would allow a better and prompt identification of the possible GAL-3-ICC false negative results. The use of GAL-3-ICC was also proposed in suspicious for malignancy thyroid nodules, in association with HBME-1, to reduce surgical risk especially in elderly patients with advanced age and co-morbidities [86]. A detailed cost-effectiveness analysis of the GAL-3-ICC is needed to compare results obtained with either GEC [94] or M/F panel [95], both characterized by a favorable cost-effectiveness profile when compared to standard of care and, in particular, to surgery. The real cost-effectiveness of the proposed algorithm also remains to be systematically analyzed.

## DISCUSSION

Preoperative characterization of thyroid nodules is one of the major problems in the clinical practice [96]. Thyroid FNA-cytology consistently improved preoperative cancer detection but the finding of indeterminate follicular lesions still represents an area of ambiguity [97]. Clinical criteria, ultrasonography as well as scintigraphy, should always be considered in the management of such nodules, but they are not accurate enough in ruling-in or ruling-out thyroid malignancies, because their unfavorable likelihood ratios [28, 98, 99]. Founded on these criteria, many unnecessary surgical procedures are still performed to remove benign lesions [29, 100–103]. The real innovation in this field, however, is the availability of new techniques, designed to identify specific genetic and epigenetic markers of thyroid malignancy. Whether their accuracy is good enough to significantly ameliorate diagnosis and treatment of such patients and, most important, to justify their cost is an open question. The AACE Thyroid scientific Committee evaluated these molecular test-methods, together with a TSHR *mRNA* blood assay, and stated that, these diagnostic approaches cannot replace the traditional clinical, US and cytopathology criteria, but integrate them [104]. The same issue was raised by the American Thyroid Association Clinical Affairs Committee, which concluded that no evidence-based recommendation could be made in favor or against the use of these methods, but they should be used with caution after a careful case-by-case evaluation [47]. A projected five-years cost-effectiveness analysis of the GEC, estimated that its translation in the clinical practice would result in a reduction of approximately 2,000 USD/patient, in a hypothetical cohort of subjects bearing indeterminate thyroid nodules, primarily because of the reduction of unnecessary diagnostic surgery [94]. Despite the large number of studies recently published on this field, especially those comparing new molecular-based test-methods characterized by rule-out versus rule-in approaches [105], there is lack of studies comparing these methods with all the others currently available, in term of diagnostic accuracy, feasibility, and most importantly in term of their cost. Our comparative analysis is based on data obtained from different populations in different settings and in different Countries and, therefore, it should not be considered as a formal comparison among cost-effectiveness analyses. Our intention was to perform a comparison of the diagnostic abilities and single test cost of competing test-methods available, all intended to ameliorate thyroid cancer detection among indeterminate thyroid nodules. Comparative analysis was performed not only using the classical indicators of sensitivity and specificity, but also using the likelihood ratios, that have been reported to be more useful [87–89]. Considering the specific features of each test a diagnostic algorithm for the clinical management of indeterminate thyroid nodules was recently proposed [31]. According to this flow chart, the GEC test-methods should be routinely used to rule-out malignancy. However, data reported in the literature and the present analysis, clearly indicate that the M/F panel + *miRNA* GEC and M/F by NGS could perform equally well in this context. In the same algorithm the use of M/F panel has been restricted to confirm malignancy in nodules suspicious for malignancy (thy4 according to BTA, tir4 according to SIE/AIT/AME/SIAPEC and category V, according to the Bethesda System), commonly referred to surgery, for a more appropriate planning of the extent of surgery (i.e. lobectomy *vs* total thyroidectomy). Although some concern has been raised regarding the ability of M/F panel to rule-out cancer, because even if the M/F panel includes the largest set of known mutations, it is possible to detect only a portion of thyroid carcinomas [106], the limitation in the use of the M/F panel appears not justified. The results of the present comparative analysis clearly indicate that M/F panel by NGS, in fact, reached an optimal results in term of sensitivity, specificity, likelihood ratios, accuracy and DOR and represents the most effective test-method that combines the ability in excluding (ruling-out) and in identifying (ruling-in) thyroid malignancy (Table 3 and Figure 4). The same result is obtained when likelihood ratios (Figure 5), accuracy (Figure 6) or DOR (Table 3) are considered. Interestingly, when the test-methods are analyzed in terms of cost and feasibility, the scenario changes. The two new molecular-based test-methods, either alone or in combination with additional molecular tests, markedly differ from the others because their costs exceed the limit of 2,000 USD (Figure 5 and Figure 6). Therefore, it seems unlikely that these methods will be used as screening test-methods in the next future, especially in low-income Countries [31]. Moreover, for optimal performance these molecular assays require centralization in a super specialized laboratory. The cost for FDG-PET/CT in Europe is approximately 1,132 USD [92]. However, it should be emphasized that this technique as well as the MIBI-Scan requires a specialized division and a trained team of experts. In addition, these exams expose the patients to potentially damaging radiations. In this regard, it has been calculated by the International Atomic Energy Agency (see IAEA, safety reports series no. 58, 2008) that the total effective dose for the whole body FDG–PET/CT averages 25 mSv, with 8 mSv due to PET and 7-30 mSv due to CT scan elements and final diagnostic scan [107]. Even if the dose can be lowered by examining only the neck, this method appears not suitable as a screening method for thyroid nodules population with indeterminate cytology. The charge for both BRAF mutation analysis and TSHR *mRNA* blood assay is effectively low, with a reported cost of 97.45 and 300 USD/test respectively. Considering their good diagnostic performance as rule-in tests, they could represent a suitable potential screening test for characterizing thyroid nodules with indeterminate cytology. However, use of BRAF mutation analysis is hampered by a rather low sensitivity. It is expressed in a fraction of papillary thyroid cancer (PTC) and in anaplastic thyroid cancer arising from PTC as well as in the follicular variant of PTC (FVPTC), but not in follicular thyroid cancer (FTC) [108, 109]. The indeterminate category is mainly constituted of FVPTC, FTC, adenomatoid hyperplasia, and follicular adenoma, all of which harbor low prevalence of BRAF mutation. It is, therefore, hard for BRAF testing to determine malignancy in this category of nodules. FVPTC and FTC may therefore represent the main source of false-negative results [110]. The published data for TSHR *mRNA* are too preliminary and are affected by some selection bias. Pooled cancer prevalence in reported studies concerning both BRAF mutation and TSHR *mRNA* were rather high (45% and 44% respectively), comparable to the highest value observed and reported in South Korea (47.4%) [9], suggesting possible selection bias (Table 1). In addition *mRNA* stability in peripheral blood may represent a limitation, which could affect the diagnostic accuracy of this specific test. There is still need for high quality validation studies before recommending the use of this procedure in large clinical settings. The clinically validated test-method with one of the lowest cost is, indeed, the GAL-3-ICC. The cost of this test-method (113 USD/test) is very competitive, compared with those estimated for FDG-PET/CT and is remarkably low compared to that reported for the molecular genetic test-methods (up to 20 times cheaper). For this reason it has been previously suggested that GAL-3-ICC could have a potential screening role, particularly in low-income Countries [31]. The present comparative analysis shows that GAL-3-ICC performs well both as an efficient rule-out and rule-in test-method, with rather good likelihood ratios and diagnostic accuracy. As previously suggested its clinical utility is, therefore, very high [111, 112]. Visualization of sensitivity, specificity, likelihood ratios, accuracy and, more importantly, cost, in both two- and three-dimensional scatterplot diagrams, clearly indicates that GAL-3-ICC represents, at the present time, the candidate test-method to be chosen on large-scale basis. Moreover, GAL-3-ICC uses conventional FNA cytological substrates, is very easy to be performed in different clinical settings and does not require to be centralized in high specialized laboratories. Recently, we demonstrated that the sensitivity of GAL-3-ICC can be further improved by combination with clinical and ultrasound follow-up of negative nodules [93]. For all these reasons GAL-3-ICC can be proposed as a screening test-method for the preoperative characterization of indeterminate thyroid nodules in different clinical settings. GAL-3-ICC was recently included in a new algorithm for the management of patients with indeterminate FNA that was based on four different markers [78]. According to this decision model the use of GAL-3-ICC was suggested in those indeterminate nodules that were negative for BRAF mutation. In addition, to further increase diagnostic accuracy, the indeterminate nodules that were negative at both BRAF mutation analysis and GAL-3-ICC were finally evaluated by real-time RT-PCR assay to detect miR-221/miR-222 expression. The proposed original sequential combination of these 4 low-cost markers may eventually lead to a better definition without increasing too much the cost. It is likely that further technical improvements of each one of the test-methods considered in this analysis, as well as the optimal combination of them, will shortly lead to a corresponding increases in the overall diagnostic accuracy and cost-effectiveness. Results of the present analysis are summarized in a proposed comprehensive algorithm (Figure 7), which differs from that previously suggested by Xing [31], because we include GAL-3-ICC as the initial screening test-method for indeterminate thyroid nodules. In case of negative test, the nodule will be monitored and eventually re-biopsied during follow-up. In those suspicious cases, in which a more accurate exclusion of malignancy is required, an additional test, with higher sensitivity/NPV (such as M/F panel by NGS and GEC) may be considered. In case of positive test, the nodule should be directly referred to surgery. However, in high-income Countries, an additional test, with higher specificity/PPV (such as the BRAF mutation analysis or M/F panel by NGS) may be suggested to confirm malignancy and confidently plane in advance the extent of surgery.

Considering that the occurrence of indeterminate thyroid nodules at conventional cytology has been reported in approximately 10-40% of FNA specimens [47], the cost saving offered by the proposed GAL-3-ICC based approach would result significant for Thyroid Centers that examine thousands of patients per year, not only in low-income countries.

## MATERIALS AND METHODS

### Literature search and study selection

We performed a systematic review and meta-analysis on the selected test-methods used for the identification of thyroid cancer in indeterminate thyroid nodules, following current protocols and guidelines [49]. A systematic search was performed on September 9, 2016, with PubMed database. We used a search query containing a combination of Medical Subject Headings (MeSH) or keywords and truncated synonyms (Boolean operators). The process of article search and selection is reported in a four-phase flow of information diagram, modified from that indicated in the PRISMA statement [49] (Figure 1). Briefly, PubMed search was initially performed by using the following search terms: “thyroid neoplasms”[MeSH Terms] OR “thyroid”[All Fields] AND “neoplasms”[All Fields] OR “thyroid neoplasms”[All Fields] OR “thyroid”[All Fields] AND “cancer”[All Fields] OR “thyroid cancer”[All Fields] OR nodule[All Fields] AND “diagnosis”[Subheading] OR “diagnosis”[All Fields] OR “diagnosis”[MeSH Terms] AND (“biomarkers”[MeSH Terms] OR “biomarkers”[All Fields] OR “marker”[All Fields] AND hasabstract[text] AND “2000/01/01”[PDAT] : “2016/12/31”[PDAT] AND English[lang]. A total of 29,307 papers were initially retrieved and then further reduced to 3,020 using the following additional search terms: “Galectin-3” [MeSH Terms], “BRAF mutation analysis” [MeSH Terms], “Gene Expression Classifier” [MeSH Terms], “FDG-PET/CT” [MeSH Terms], MIBI scan [MeSH Terms], “TSHR *mRNA*” [MeSH Terms] and “mutation/fusion panel” [MeSH Terms]. For each test-method selected we retrieved available information regarding the cost of a single test, as reported below. In particular, 259 papers were retrieved using the term “Galectin-3”, 1,060 with “BRAF mutation analysis”, 64 with “Gene Expression Classifier”, 839 with “Mutation/fusion panel”, 596 with “FDG-PET/CT”, 156 with “MIBI-scan” and 46 using the term “TSHR *mRNA*”. Subsequently, another selection criteria was applied using the term “Fine Needle Aspiration Cytology” that reduced the number of papers down to 83 for Galectin-3-ICC, to 224 for “BRAF mutation analysis”, to 44 for “Gene Expression Classifier”, to 143 for “Mutation Fusion Panel”, to 88 for “FDG-PET/CT”, to 26 for “MIBI-scan” and to 5 for “TSHR *mRNA*”. Finally, our comparative analysis was performed on those studies that, were chosen according to the following inclusion criteria: a) thyroid nodule population examined that was classified as thy3a/thy3f/tir3A/tir3B/III/IV/indeterminate thyroid nodules; b) studies that were based on more than 50 indeterminate thyroid lesions; c) studies in which all examined patients were surgically treated and cytologic reports as well as the results of the different test-methods used were verified at the final histology; d) validation studies in which patients were recruited either in one single or in more than one clinical center. Conversely, studies showing the following exclusion criteria were not considered in this analysis: a) thyroid nodule population examined that was classified as suspicious for malignancy (thy4/tir4/V), unequivocal cancer at cytology (thy5/tir5/VI) and follicular tumors with undefined malignant potential (FTUMP); b) studies that were based on less than 50 cases; c) post-validation studies in which not all patients were surgically treated and/or the final histology was lacking; d) studies in which the exact number of true and false positive and negative cases wasn't clearly reported. No specific data have been published yet regarding the diagnostic accuracy of anyone of the different test-methods examined in the new recently proposed nosological thyroid cancer entity, named “encapsulated follicular variant of papillary thyroid carcinoma” (EFVPTC) [113] that was not included in the study. By adopting such criteria we were able to select a total of 9 papers for GAL-3-ICC, 24 for BRAF mutation analysis, 2 for GEC, 9 for M/F panel, 1 for FDG-PET/CT, 3 for MIBI-Scan and 2 for TSHR *mRNA*. Study eligibility and quality appraisal of retrieved full-text articles were all evaluated and graded independently by 2 investigators. Discrepancies were resolved by consensus. All these selected studies were included in the present comparative analysis.

### Analysis of study heterogeneity

The possible occurrence of systematic heterogeneity was evaluated in the four test-methods subjected to meta-analysis using the forest plot. In order to use the appropriate method significance of heterogeneity was preliminary assessed for each method and fixed effect model, according to the method of Mantel and Haenszel [114] was used for the analysis of BRAF mutation and MIBI-Scan selected studies, while random effect model, using the method of DerSimonian and Laird [114], was applied for the analysis of GAL3-ICC and M/F panel selected studies. The estimate of heterogeneity was obtained using the Mantel–Haenszel model [114]. The software used for meta-analysis was the Stata Statistical Software (Release 12, 2011, StataCorp LP, College Station, TX) [115, 116].

### Analysis of diagnostic test accuracy

Diagnostic performances of the seven different test-methods considered in the analysis were evaluated by applying the basic 2-by-2-table for estimating the diagnostic accuracy of a dichotomous or dichotomized quantitative test result. Patients with follicular thyroid proliferations with and without atypia as well as Hürthle cell follicular proliferations were included in the calculations, while follicular tumors with undefined malignant potential (FT-UMP) were excluded from statistical analyses because they remain indeterminate also at final histology. For each reported test-method sensitivity, specificity, PPV, NPV, FPR, FNR, FDR, LR+ and LR-, Cancer risk rate in positive test and Cancer risk rate in negative test were calculated, as well as three measures of diagnostic test accuracy, namely accuracy, F1 score and DOR. The following formulas were used for calculations: sensitivity or true positive rate (TPR)=TP/(TP+FN), negative predictive value (NPV)=TN/(TN+FN), cancer risk in negatives test=FN/(FN+TN), false negative rate (FNR)=1-TPR, negative likelihood ratio (LR-)=(FN/(TP+FN))/(TN/(FP+TN)), specificity or true negative rate (TNR)=TN/(TN+FP), cancer risk in positives test or positive predictive value (PPV)=TP/(TP+FP), false positive rate (FPR)=1-TNR, positive likelihood ratio (LR+)=(TP/(TP+FN))/(FP/(FP+TN)), accuracy=(TP+TN)/(P+N), F1 score=2TP/(2TP+FP+FN), and diagnostic odds ratio (DOR)=(TP/FP)/(FN/TN). The diagnostic performance values of all studies included in the meta-analyses were pooled together.

### Comparative two- and three-dimensional scatterplot diagrams

In order to comparatively evaluate accuracy, sensitivity, specificity and costs of each test-method two- and three-dimensional scatterplot diagrams were analyzed and visualized using the Stata Statistical Software (Release 12, 2011, StataCorp LP, College Station, TX) [115, 116] and the online visualization option from Plotly [117].

### Reported cost of each test-method

A detailed cost effectiveness analysis is available for only few methods, while for the others we are able to report only the cost of each single test. In particular, for Gal-3-ICC a detailed study regarding its cost efficacy has not been performed so far. The cost of a single Gal-3-ICC test may change in the different context but, as an average in Italy, it can be estimated as 100 euro, corresponding to 113 USD (1 USD = 1.1306 EUR) [90]. A projected five-years cost-effectiveness analysis of the GEC was published in 2011 [94]. According to this study and to another recently published study [118], the base case cost of this test-method is 3,200 USD. The reported cost of BRAF mutation analysis is 475 USD [118, 119]. Combination of the cost of this test together with the cost of the GEC alone, gives a total price of 3,675 USD. The BRAF medical cost is USD 97,45 (range 55-123) according to published studies as well as Medicare reimbursement rate [95, 120]. A detailed cost-effectiveness analysis of M/F panel applied to FNA samples was performed and published [121]. The single test cost is 2,250 USD. Medicare reimbursement is currently 650 USD, while private reimbursement is 950 USD [94]. The cost of a single test using the M/F panel by NGS was reported to be 3,200 USD [121]. The Medicare reimbursement for the TSHR *mRNA* test is 300 USD [121]. To date a cost efficacy analysis for this method has not been published yet. An accurate cost-effectiveness analysis of FDG-PET/CT was recently published [92]. The mean cost of a single FDG-PET/CT in the Netherlands was 1,002 EUR [121], equivalent to 1,132 USD/test [90] and is similar to that reported in Great Britain [122] and in Germany [123]. In the United States, the Medicare program provides reimbursement for PET and PET/CT. In particular, for examinations performed on inpatients or at hospital outpatient departments, a median amount of 952.83 USD is reimbursed. The cost as well as the radiation exposure, expressed in terms of millisievert (mSv), can be lowered if the scan is restricted to the neck and focused to the thyroid bed. The reported mean cost of a single MIBI-Scan in Germany was 1,459 EUR [48], equivalent to 1,648 USD/test [90].
